# Reduced Perceived Trustworthiness during Face Mask Wearing

**DOI:** 10.3390/ejihpe11040105

**Published:** 2021-11-19

**Authors:** Giulio Gabrieli, Gianluca Esposito

**Affiliations:** 1 Psychology Program, Nanyang Technological University, Singapore 639818, Singapore; giulio001@e.ntu.edu.sg; 2 Department of Psychology and Cognitive Science, University of Trento, 38068 Trento, Italy; 3 Lee Kong Chian School of Medicine, Nanyang Technological University, Singapore 308232, Singapore

**Keywords:** halo effect, aesthetic appearance, trustworthiness, face mask

## Abstract

To curb the diffusion of the novel coronavirus (SARS-CoV-2), governments worldwide have introduced different policies, including lockdowns, social distancing, and mandatory mask wearing. Face mask wearing, especially, has an impact on the formation of first impressions, given that when meeting someone for the first time, individuals rely on the only available piece of information, the newly met person’s aesthetic appearance, in order to make initial estimations of other traits, such as competence, intelligence, or trustworthiness. However, face mask wearing affects the aesthetic appearance of an individual, creating uncertainty which, in turn, has been reported to reduce others’ perceived trustworthiness. In this paper, the influence of face mask wearing on strangers’ perceived trustworthiness and aesthetic appearance is assessed to verify the impact of this policy on impression formation. Participants (N = 71) have been instructed to assess the trustworthiness and the aesthetic appearance of a selection of 96 images depicting individuals of different ages (children, adults, and older adults), gender (men and women), and ethnicity (Asians or Caucasians). Participants were randomly divided into two groups: an experimental group and a control group. Participants in the experimental group (N = 38) rated faces of individuals wearing a face mask, while participants in the control group rated the same faces but in the absence of a face mask. Images were presented in random order. For each face, participants were asked to rate the aesthetic appearance and perceived trustworthiness of the stranger on two different 100-point Likert scales. Results demonstrate that (i) the correlation between perceived trustworthiness and aesthetic appearance is not affected by the presence of a face mask, and (ii) age, but not ethnicity and gender, influences the magnitude of differences in perceived trustworthiness levels during mask wearing.

## 1. Introduction

Faces are known to play a prominent role in social cognition [1,2], being stimuli from which it is possible to estimate different traits of a person. It is possible to extract several objective characteristics, such as ethnicity, gender or age, of a stranger from the aesthetic appearance of the face. However, humans have a natural inclination to anticipate other non-directly measurable attributes, such as intelligence, competence, or trustworthiness, based on the attractiveness of the face.

Previous works demonstrated the existence of a correlation between the “aesthetic appearance”, which refers to the objective judgment of the appearance, and the “perceived trustworthiness” of an object or an individual, which refers to the level of confidence in avoiding, approaching, or interacting with the person or the object [3]. When meeting someone for the first time, the only information available to make inferences about their trustworthiness is their aesthetic appearance. The influence of the aesthetic appearance on other traits, such as intelligence [4,5], warmth [6], or trustworthiness, [7,8] has been defined as the halo effect. Usually measured as the correlation between aesthetic and a second trait, past works demonstrated the existence of a positive halo effect, such that more aesthetically pleasing individuals are also expected to be more intelligent, warm, or trustworthy.

The relationship between perceived trustworthiness and aesthetic appearance gained special interest in social psychology, being one of the core factors that guide the interaction between two strangers. Since the first half of the twentieth century, several empirical investigations explored the association between aesthetic appearance and perceived trustworthiness [6,9]. Confirming the stereotype of “what is beautiful is good” [10], a significant positive association between aesthetic and perceived trustworthiness has been demonstrated (for a review see [11]).

Throughout the last two years, national authorities have implemented laws and regulations aimed at limiting the propagation of the new coronavirus, including but not confined to flying bans, movement control restrictions, social distance, and the compulsory use of face masks. Although the implications of such initiatives on individual citizens’ mental well-being and loneliness has been researched [12,13], limited research focused on the influence of alteration in aesthetic appearance—caused by face mask use—on the halo effect, particularly the linkage between aesthetic appearance and perceived trustworthiness. Despite the fact that the practice of face mask wearing helped reduce the diffusion of the SARS-CoV-2 [14,15], wearing a mask obscures many of the visual clues that are frequently used in social interactions to assess the psychological states and intents of others [16,17]. Additionally, the same facial characteristics are used to form trait evaluations, such as perceived trustworthiness [18,19]. When portions of the face are covered, as when forced to wear a surgical mask, context clues that are ordinarily used to predict attributes are completely invisible, establishing a sense of insecurity that impacts social judgments [20]. The feeling of uncertainty has been shown to reduce perceptions of likability, trustworthiness, and closeness [21,22,23].

A recent analysis by Grundmann et al. [24] investigated the effect of mask wearing on social judgments and emotion recognition, such as trustworthiness. In their study, 191 German adults, both men and women, judged photos of other German adults. Despite Grundmann et al. [24] predicting that wearing a facial mask would reduce perceived trustworthiness, the results did not support the hypothesis. In fact, using a face mask had no effect on perceived trustworthiness on its own. The findings of Grundmann et al. [24] corroborate the findings of Cartaud et al. [25], which revealed that wearing a face mask boosted strangers’ perceived trustworthiness.

Despite its indubitable validity, the study conducted by Grundmann et al. [24] has a major limitation, which is that it exclusively involved German participants who rated only adult faces of the same ethnicity. As a result, it is hard to assert that the effect [26,27] is generalizable to other populations. Previous research investigated the universal applicability of the halo effect between aesthetic appearance and perceived trustworthiness across age groups, genders, and ethnicities, indicating that age, but not gender or ethnicity, increases the strength of the halo [8,28,29]. However, these studies employed faces in which all the visual cues were available for the rater.

### Aim and Hypotheses

The aim of this work is to explore the influence of the practice of face mask wearing on perceived trustworthiness of strangers’ faces and how ethnicity, gender, and age influence changes in trustworthiness when face masks are worn. Based on the results of previous studies, two hypotheses are formulated and preregistered [30]:

**Hypothesis** **1.** *The strength of the correlation between aesthetic appearance and perceived trustworthiness is influenced by the presence of a face mask on the face itself, as well as by the age, gender (same vs. different from participant), and ethnicity (same vs. different from participant) of the presented face. A stronger correlation between aesthetic appearance and perceived trustworthiness is expected in the non-mask condition, as well as for adult faces of the same gender and ethnicity as the participant.*

Rationale:

When faces are completely visible, inferences about strangers’ trustworthiness can be made from the combinations of all the different elements of the face. However, when parts of the face are covered, only a limited amount of visual information is available, increasing the uncertainty towards the stranger [21]. Therefore, assuming that the perceived trustworthiness is estimated from the aesthetic appearance, greater uncertainty could result in greater variability in collected measures, with the subsequent reduction in correlation between aesthetic appearance and perceived trustworthiness, which is the measure of halo. However, this may not be true for faces of different age groups. In fact, Collova et al. [31] tested the signal threat response of both children’s and adults’ faces on Oosterhof and Todorov [32]’s two-dimensional model (dominance and trustworthiness), confirming differences in the estimation of trustworthiness of adults’ and children’s faces. More specifically, adults’ judgment of children’s perceived trustworthiness may not rely on their aesthetic appearance as much as it may with other adults’ faces. This possibility was confirmed by multiple studies on the halo effect, which demonstrated that the halo between aesthetic appearance and perceived trustworthiness is lower for children’s, as compared to adults’ faces [8,28]. Hence, differences in the halo between the mask wearing and control conditions are expected for adults’ but not for children’s faces, with a lower correlation between aesthetic appearance and perceived trustworthiness for adults’ faces in the mask-wearing condition. For what concerns the ethnicity and gender of other adults, controversial results have been found in previous works, with articles reporting a stronger halo for individuals of the same gender and ethnicity [6], while other studies have found no significant effect of either the gender or ethnicity on the strength of the halo [8,28,29,33]. To confirm the results of Carter [6], we hypothesized the existence of a stronger halo for adults of the same gender and ethnicity as the rater.

**Hypothesis** **2.** *The judgments of perceived trustworthiness are influenced by the presence of a mask, as well as by the age, gender (same vs. different from participant), and ethnicity (same vs. different from participant) of the presented face. Lower trustworthiness is predicted in the mask condition, while a higher trust for children’s faces, as well as for faces of participants’ with the same gender and ethnicity.*

Rationale:

As reported for the previous hypothesis (H1), limiting the amount of information about a face increases the uncertainty toward the stranger [21]. Uncertainty has been demonstrated to have a negative influence on perceived trustworthiness [21,24,34,35]. As such, lower perceived trustworthiness is expected for faces in the mask-wearing condition as compared to the control condition. However, if children’s faces are a special class of stimuli for which perceived trustworthiness is less influenced by the aesthetic appearance [1,31], the differences in the trustworthiness of children’s faces between the control and face mask condition should be lower than the differences that are expected for adults’ faces. Moreover, according to the ingroup bias theory [36,37], preference should be given to individuals of similar age, gender, and ethnicity; therefore, a smaller change in perceived trustworthiness is expected for the ingroup’s adults, as opposed to adults belonging to the outgroup (different gender, different ethnicity). Therefore, lower perceived trustworthiness is expected for adults in the mask-wearing condition of different ethnicity and gender as compared to the participant’s gender.

## 2. Materials and Methods

### 2.1. Experimental Design

The research methodology is based on the paradigm used in prior research on the halo effect by Gabrieli et al. [8,28,29]. Participants were randomly assigned to one of two groups after enrolling in the study: a control group (with faces presented without face masks) and an experimental group (with faces presented wearing a face mask). Participants were asked to judge the perceived trustworthiness and aesthetic appearance of each face, using two 100-point Likert scales—where 1 stands for “Not at all” and 100 for “Extremely”—of 96 images, presented in random order.

As the aim of the study was to test the influence of age, gender, and ethnicity, three different age groups (child, adult, or older adult), two genders (man or woman), and two ethnicities (Asian and Caucasian) were targeted. In order to be selected, images had to portray a single individual, front facing, with no visible disturbing elements that cover parts of the face (e.g., hat, face mask, sunglasses). For each possible combination of age, gender, and ethnicity, 8 images were selected from the FFHQ dataset, for a total of 96 images. The FFHQ dataset contains 70,000 high-quality (1024 × 1024) pictures of faces posted on Flickr (https://www.flickr.com, accessed on 17 November 2021), an online photo storage and file-sharing site licensed under several creative commons and public domain agreements (U.S. Government Works license, Creative Commons BY-NC 2.0, Creative Commons BY 2.0, and Public Domain Mark 1.0, Public Domain CC0 1.0), and has been employed in previous studies on the halo effect [38,39]. The same 96 images selected for the study here reported were previously employed in other studies on the halo effect [28,29]. This set of 96 images was presented to participants in the control group. To generate the stimuli for the experimental group, GIMP—an open-source photo manipulation tool—was used in order to add surgical face masks on presented faces. The same surgical face mask was placed on all 96 faces. The whole set of employed images can be found online on the repository of this study: https://doi.org/10.21979/N9/KOAPLW (accessed on 17 November 2021).

### 2.2. Analytic Plan

To test the two proposed hypotheses, two separate analysis of variance (ANOVA) were employed:

(1) Halo=Age×Gender×Ethnicity×Condition

(2) Trustworthiness=Ethnicity×Gender×Age×Condition

where halo is operationally defined as the correlation (Pearson’s correlation) between aesthetic appearance and perceived trustworthiness.

To estimate the required sample size, a power analysis was performed in G*Power [40,41]. Given the number of hypotheses, a correction for multiple comparisons has been applied (Bonferroni’s correction, corrected alpha = 0.025). For the type of tests (between–within subjects ANOVA), corrected alpha value, and predicted effect size f (f = 0.15, estimated from [8]), in order to achieve a high statistical power (0.95), at least 70 participants were required. The analytic plan was preregistered on the Open Science Framework prior to the data collection [30].

### 2.3. Participants

Participants were recruited via advertisement on social media, the Reddit community r/samplesize, the online research participation system of the School of Social Sciences (Nanyang Technological University, Singapore), and the online platform Surveycircle. Data collection took place between August and September 2021. Participants received no monetary reimbursement; however, participants enrolled at the School of Social Sciences (Nanyang Technological University, Singapore) received university credits for their participation.

The experimental paradigm was implemented in Qualtrics. Participants completed the survey with their own devices remotely. Completion took on average 40.41 ± 23.35 min.

Seventy-three (N = 73) adults completed the survey. In order to better investigate the role of gender, data from two (N = 2) participants who preferred not to reveal their gender were excluded. Therefore, the final sample constitutes seventy-one (N = 71, mean age = 26.44 ± 8.27) participants. Of this total, 44 identified as women, while 33 as men. For what concerns the division in the control and experimental groups, thirty-three (N = 33) participants were randomly assigned to the control group, while thirty-eight (N = 38) to the experimental condition. Participants’ demographic information by ethnicity and gender are reported in Table 1.

## 3. Results

A summary of the first ANOVA (Equation (1)) is reported in Table 2. Concerning the first hypothesis (H1)—which regarded the influence of ethnicity, gender, age, and condition on the strength of the halo effect—the results shown in Table 2 reveal the absence of significant main and interaction effects for the investigated variables. The absence of any difference in the strength of the halo, measured as the Pearson’s correlation between perceived trustworthiness and aesthetic appearance, was further confirmed by means of a post hoc test that compared the correlation between the two variables by condition (Fisher’s z = −0.3288, *p*-value = 0.7423).

**Figure 1:**
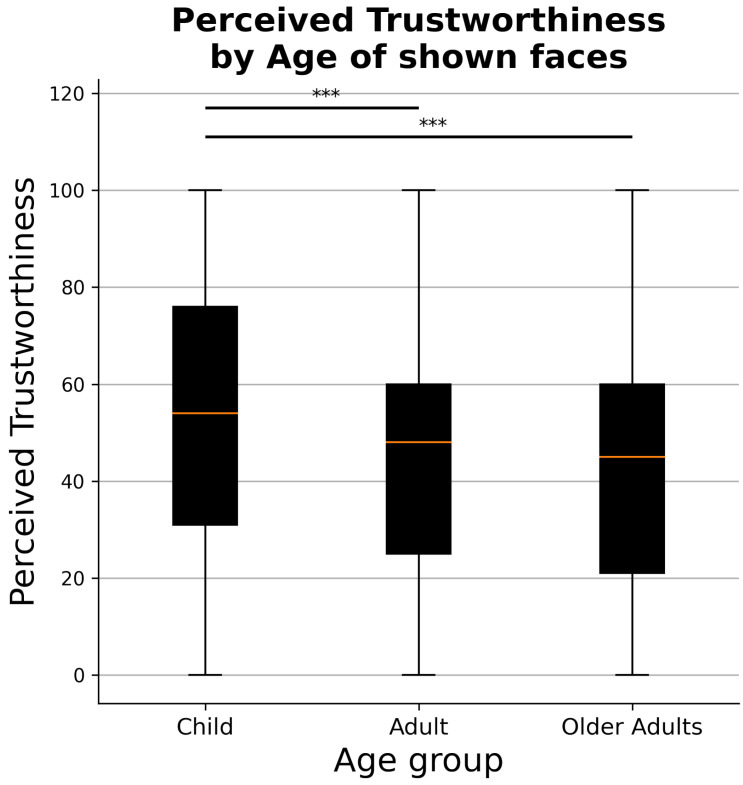
Perceived trustworthiness judgments by age group of presented faces. *** *p* < 0.001.

Focusing on the second hypothesis (H2)—that focuses on differences in perceived trustworthiness—results of the ANOVA, reported in Table 3, highlight the existence of a significant main effect of the variables age, gender, and condition. Moreover, significant interaction effects between age and gender and between age and condition were found. Post hoc analysis confirmed that children’s faces were rated higher in perceived trustworthiness, as opposed to adults’ (Mann–Whitney U = 3,127,237.5, *p*-value = 4.414 $\times 10^{-35}$) and older adults’ faces (Mann–Whitney U = 3,190,112.0, *p*-value = 3.379$\times 10^{-43}$, Figure 1). No significant differences in trustworthiness judgments were found for what concerns the scores assigned to adults’ and older adults’ faces (Mann–Whitney U = 2,661,147.0, *p*-value = 0.070). Additionally, a post hoc analysis revealed a preference for same-gender faces (mean perceived trustworthiness = 48.25 ± 26.40), as opposed to different-gender faces (mean perceived trustworthiness = 45.99 ± 27.19; Mann–Whitney U = 6,119,924.5, *p*-value = 0.000118). Moreover, a paired t-test post hoc comparison demonstrated that faces with a face mask received significantly lower perceived trustworthiness evaluations (mean perceived trustworthiness = 44.57 ± 6.72), as compared to when shown without a face mask (mean perceived trustworthiness = 50.05 ± 9.20; *t*-value = 12.619, *p*-value = 5.060$\times 10^{-22}$).

**Figure 2:**
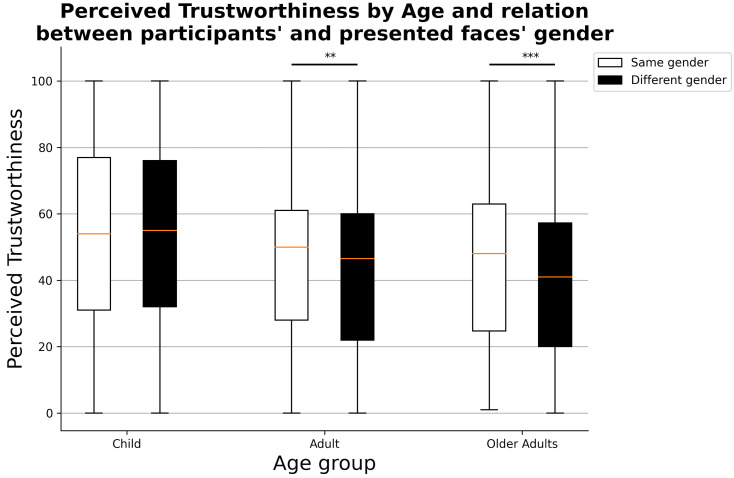
Perceived trustworthiness judgments by age group of presented faces and relationship between participant’s and face’s gender. ** *p* < 0.01, *** *p* < 0.001.

**Figure 3:**
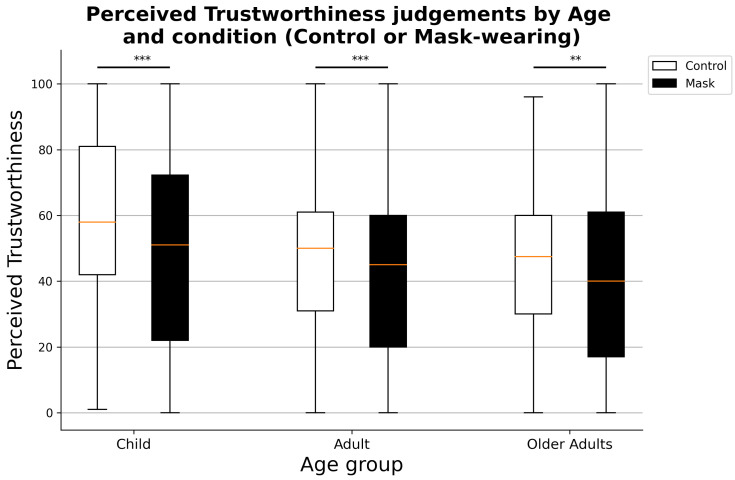
Perceived trustworthiness judgments by age group of presented faces and condition. ** *p* < 0.01, *** *p* < 0.001.

Focusing on the interaction effects, no differences are present between same- and different-gender children’s faces (Mann–Whitney U = 644031.5, *p*-value = 0.938), while both adults’ and older adults’ same-gender faces received significantly higher perceived trustworthiness scores than different-gender faces (Mann–Whitney U = 715,701.0, *p*-value = 6.575$\times 10^{-6}$, Figure 2). For what concerns the interaction between the condition and the age of presented faces, significantly higher perceived trustworthiness judgments were assigned to faces in the control condition (non-masked) for all three age groups (child: Mann–Whitney U = 741,231.0, *p*-value = 1.968$\times 10^{-10}$; adults: Mann–Whitney U = 716,153.5, *p*-value = 2.005$\times 10^{-6}$; older adults: Mann–Whitney U = 703,136.5, *p*-value = 8.941$\times 10^{-5}$, Figure 3).

## 4. Discussion

The current study analyzed the influence of donning a face mask on others’ perceived trustworthiness, as well as the halo effect between aesthetic appearance and perceived trustworthiness. The preregistered expectations were only partially supported by the results.

Pertaining to the first Hypothesis (H1), which looked at the influence of ethnicity, gender, age, and condition on the strength of the halo effect, results of the ANOVA (Table 2) did not support the initial prediction. In fact, we predicted the halo effect to be stronger in the control condition, as well as for same-gender and same-ethnicity adults’ faces. However, neither significant main nor interaction effects were found. Although the lack of a significant effect for the gender and ethnicity factors is not unexpected, it is consistent with earlier observations on the halo effect. The lack of a significant main effect of the condition and age of the shown faces was more striking. With regards to the condition, in the absence of a face mask, because the viewer has access to more visual elements and the evaluation of trustworthiness of a stranger was assumed to be dependent on the amount of visual cues available, a stronger halo was anticipated. The halo was observed to be relatively stronger in the control condition, but the difference in magnitude between the two conditions did not exceed the significance threshold. Further analysis using a two-tailed paired *t*-test found that faces in the experimental condition were not only rated as less trustworthy (*t*-value = 12.619, *p*-value = 5.060$\times 10^{-22}$) but also as less aesthetically pleasing, as compared to when the same faces were shown in the control condition (*t*-value = 10.35, *p*-value = 3.025$\times 10^{-17}$). Consequently, the strength of the halo effect is unaffected by the volume of visual elements available to make a trustworthiness estimation. When face masks are worn, the perceived aesthetic appearance of a face decreases, which leads to a decrease in trustworthiness. This is consistent with what is already known in the literature about the halo effect that exists between aesthetic appearance and perceived trustworthiness.

As a whole, contrary to the results of Cartaud et al. [25], the findings validate the observations of Grundmann et al. [24], which found a reduction in perceived trustworthiness upon wearing a face mask. What underpins the decrease is discussed here in terms of a diminished experience of aesthetic appearance, which results in lower perceived trustworthiness due to a sustained halo between appearance and trustworthiness of faces, irrespective of the amount of visual cues made accessible to the viewer.

The absence of a significant main effect of age, as well as the absence of an interaction effect between age and condition, is likely due to the present study’s limited sample. Indeed, prior evidence on the halo effect was used to estimate the sample size needed for this present investigation [8,28]. Nevertheless, previous studies’ estimations could be larger than the true effect size, and thus the calculated sample size would be too limited to reach sufficient statistical power. Alternatively, the strength of the halo effect varies over time, and thus the contribution of the diverse visual cues may vary correspondingly. As per the results presented in Table 2, the estimated $\eta_{p}^{2}$ for age is $1.37\times 10^{-3}$, which equates to an effect size *f* of about 0.04. This effect size is smaller than the effect size that was used in the power analysis. Although we cannot rule out the possibility that the age of presented faces does not influence the magnitude of the halo effect linking aesthetic appearance and perceived trustworthiness, a more plausible explanation is that the sample size targeted for this current study is not sufficient to detect the effect.

The results of the ANOVA (Table 3) partially confirm the predictions of the second hypothesis pertaining to variations in perceived trustworthiness. The findings confirm the expectation that the use of a face mask diminishes a stranger’s assessed level of trustworthiness and that age and gender influence the formation of perceived trustworthiness, while gender has no impact. More precisely, there were no disparities in the perceived trustworthiness of same-gender versus different-gender children’s faces; however, same-gender adults and older adults had greater trustworthiness scores than different-gender faces. Despite the fact that a preference for non-masked faces was, as expected, found for all three tested age groups ($\eta_{p}^{2}=0.04$, effect size f = 0.20, achieved power = 0.99), the small effect of gender ($\eta_{p}^{2}=1.87\times 10^{-3}$, effect size f = 0.04, achieved power = 0.07) was not expected in light of previous results published in the literature.

Overall, the findings in this paper offer a number of intriguing insights into the halo effect between aesthetic appearance and perceived trustworthiness, as well as the former of the two variables when visual cues of a face are partially hidden by some kind of visual obstruction, such as a surgical mask. The findings of this study suggest that the halo effect is unaffected by the portion of visual information needed to make assumptions about a stranger’s trustworthiness, but the level of perceived trustworthiness is affected by the age and gender of a stranger’s face, though not by ethnicity. Furthermore, the involvement of gender appears to play a role only for adults’ and older adults’ faces but not for children’s faces, supporting the notion that children’s faces are uniquely processed. Notwithstanding, unlike previous studies, the age of the unobstructed face had no impact on the strength of the halo effect in the present research, most likely due to the relatively small sample size, which may have been overestimated prior to data collection. Nonetheless, the findings presented here are, to the best of our knowledge, among the first to shed light on the impact of changes in a stranger’s aesthetic appearance induced by mask wearing on his or her perceived trustworthiness.

### Limitations

The study presented here has some limitations. First, while the required sample size was estimated accordingly to the results of previous works on the halo effect between aesthetic appearance and perceived trustworthiness, in the current study the possible effect size, estimated from Table 2’s $\eta_{p}^{2}$, seems to be lower. As such, one possibility is that the current work does not have sufficient statistical power to correctly identify the effect. In fact, a sensitivity analysis confirms that the minimum effect size detectable with the current sample size is 0.1478 (α = 0.025, power = 0.95, $\eta_{p}^{2}$ = 0.02), while for an effect size f of 0.037 (estimated from $\eta_{p}^{2}$ = 1.37$\times 10^{-3}$) the achieved power would be far below the acceptability threshold. Future works should replicate the paradigm here reported on a larger sample in order to have sufficient power to reliably test the influence of age on the halo effect during face mask wearing.

Moreover, the study employs a limited number of faces (N = 96). While the number of stimuli was selected for consistency with past works, the number of stimuli is unable to cover the infinite possible characteristics of the human face. As such, future works should include different stimuli to test the generalization of the findings here presented to different faces.

Additionally, only three broad age groups, two genders, and two ethnicities have been considered in the study here reported. Future replications of the current study should verify the generalizability of the findings to individuals who do not identify as either man or woman, individuals of different ethnicities, and may consider investigating the effects on more fine-tuned age groups.

## 5. Conclusions

In this paper, the influence of changing the aesthetic appearance of a stranger’s face on the perceived trustworthiness and on the halo effect between the two measures has been discussed.

Observations revealed that age and gender of strangers’ faces, but not their ethnicity, have an influence on the reduction in perceived trustworthiness during face mask wearing. Adult and older adult individuals of different gender are perceived as significantly less trustworthy when wearing a face mask, as compared to when their full faces are visible. Results of this current work may help shed light on the consequences of having part of the face covered during the formation of first impressions. Policymakers should consider how the practice of face mask wearing affects the perception of trustworthiness of strangers, especially in cases in which trusting strangers is crucial.

## Figures and Tables

**Table 1:** Mean age (standard deviation) of enrolled participants by gender and ethnicity.

Ethnicity	Gender	N	Mean Age (±std)
Caucasian	Men	9	28.33 ± 7.00
Caucasian	Women	26	30.27 ± 10.11
Asian	Men	22	22.05 ± 2.73
Asian	Women	14	25.00 ± 7.93

**Table 2:** Summary of the ANOVA model (Equation (1)).

	Sum Sq	Df	F Value	*p*	$\eta_{p}^{2}$
Ethnicity	0.008	1	0.0462	0.8299	5.84 $\times \;10^{-5}$
Age	0.196	2	0.5428	0.5813	1.37 $\times \;10^{-3}$
Gender	0.254	1	1.4050	0.2362	1.77 $\times \;10^{-3}$
Condition	0.076	1	0.4172	0.5185	5.27 $\times \;10^{-4}$
Gender × Age	0.523	2	1.4437	0.2367	3.64 $\times \;10^{-3}$
Ethnicity × Age	0.464	2	1.2828	0.2778	3.23 $\times \;10^{-3}$
Gender × Ethnicity	0.057	1	0.3124	0.5764	3.95 $\times \;10^{-4}$
Age × Condition	0.500	2	1.3823	0.2516	3.48 $\times \;10^{-3}$
Gender × Condition	0.004	1	0.0228	0.8800	2.88 $\times \;10^{-5}$
Ethnicity × Condition	0.003	1	0.0170	0.8962	2.15 $\times \;10^{-5}$
Ethnicity × Gender × Age	0.397	2	1.0970	0.3344	2.77$\times \;10^{-3}$
Gender × Age × Condition	0.590	2	1.6304	0.1965	4.11 $\times \;10^{-3}$
Ethnicity × Age × Condition	0.146	2	0.4044	0.6675	1.02 $\times \;10^{-3}$
Ethnicity × Gender × Condition	0.106	1	0.5834	0.4452	7.37 $\times \;10^{-4}$
Ethnicity × Gender × Age × Condition	0.199	2	0.5500	0.5772	1.39 $\times \;10^{-3}$

**Table 3:** Summary of the ANOVA model (Equation (2)).

	Sum Sq	Df	F value	*p*	$\eta_{p}^{2}$
Ethnicity	503	1	0.7378	0.3904	1.09 $\times \;10^{-4}$
Age	190,887	2	139.9388	<0.0001 ***	0.04
Gender	8699	1	12.7539	0.0004 ***	1.87 $\times \;10^{-3}$
Condition	51,064	1	74.8705	<0.0001 ***	0.01
Age × Gender	5545	2	4.0650	0.01720 *	1.20 $\times \;10^{-3}$
Age × Ethnicity	996	2	0.7302	0.4818	2.15 $\times \;10^{-4}$
Gender × Ethnicity	569	1	0.8348	0.3609	1.23 $\times \;10^{-4}$
Age × Condition	7674	2	5.6257	0.0036 **	1.65 $\times \;10^{-3}$
Gender × Condition	2	1	0.0023	0.9621	3.33 $\times \;10^{-7}$
Ethnicity × Condition	180	1	0.2646	0.6070	3.90 $\times \;10^{-5}$
Ethnicity × Gender × Age	151	2	0.1108	0.8951	3.26 $\times \;10^{-5}$
Gender × Age × Condition	988	2	0.7246	0.4846	2.13 $\times \;10^{-4}$
Ethnicity × Age × Condition	989	2	0.7253	0.4842	2.14 $\times \;10^{-4}$
Ethnicity × Gender × Condition	111	1	0.1626	0.6868	2.39 $\times \;10^{-5}$
Ethnicity × Gender × Age × Condition	416	2	0.3051	0.7370	8.98 $\times \;10^{-5}$

* *p* < 0.05, ** *p* < 0.01, *** *p* < 0.001.

## Data Availability

The dataset generated for this publication, as well as the scripts employed for the analysis, are available online at the following page: https://doi.org/10.21979/N9/KOAPLW (accessed on 17 November 2021).
